# Supplementary material for: Please Mind the Gap: Indel-Aware Parsimony for Fast and Accurate Ancestral Sequence Reconstruction and Multiple Sequence Alignment Including Long Indels
Source: Mol Biol Evol. 2024 Jun 6;41(7):msae109. doi: 10.1093/molbev/msae109 (PMC11221656; doi:10.1093/molbev/msae109)
Supplement: msae109_Supplementary_Data [file msae109_supplementary_data.pdf]

Supplementary Material

Ancestral sequence reconstruction substitution, insertion and deletion error for tree height 0.8

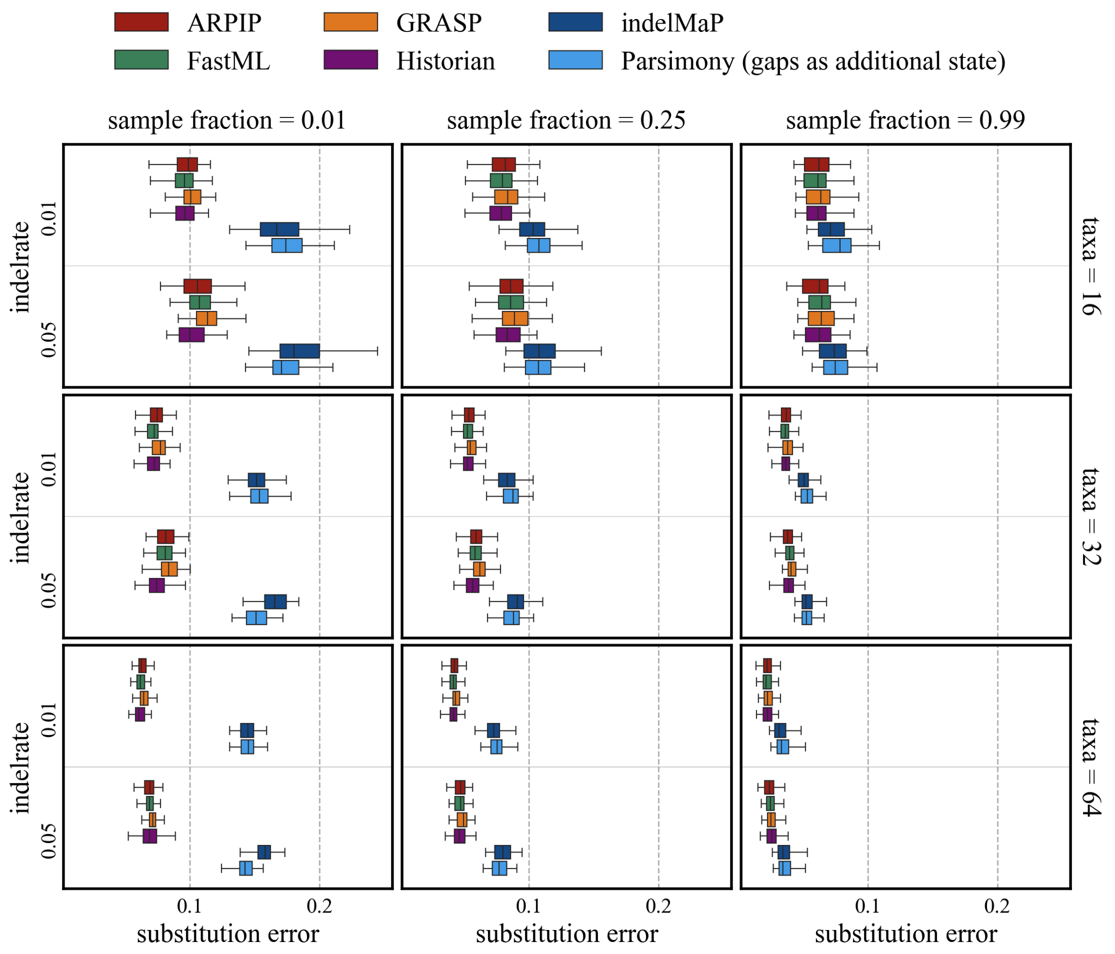

Figure S1: Substitution error for all parameter combinations for trees with tree height 0.8

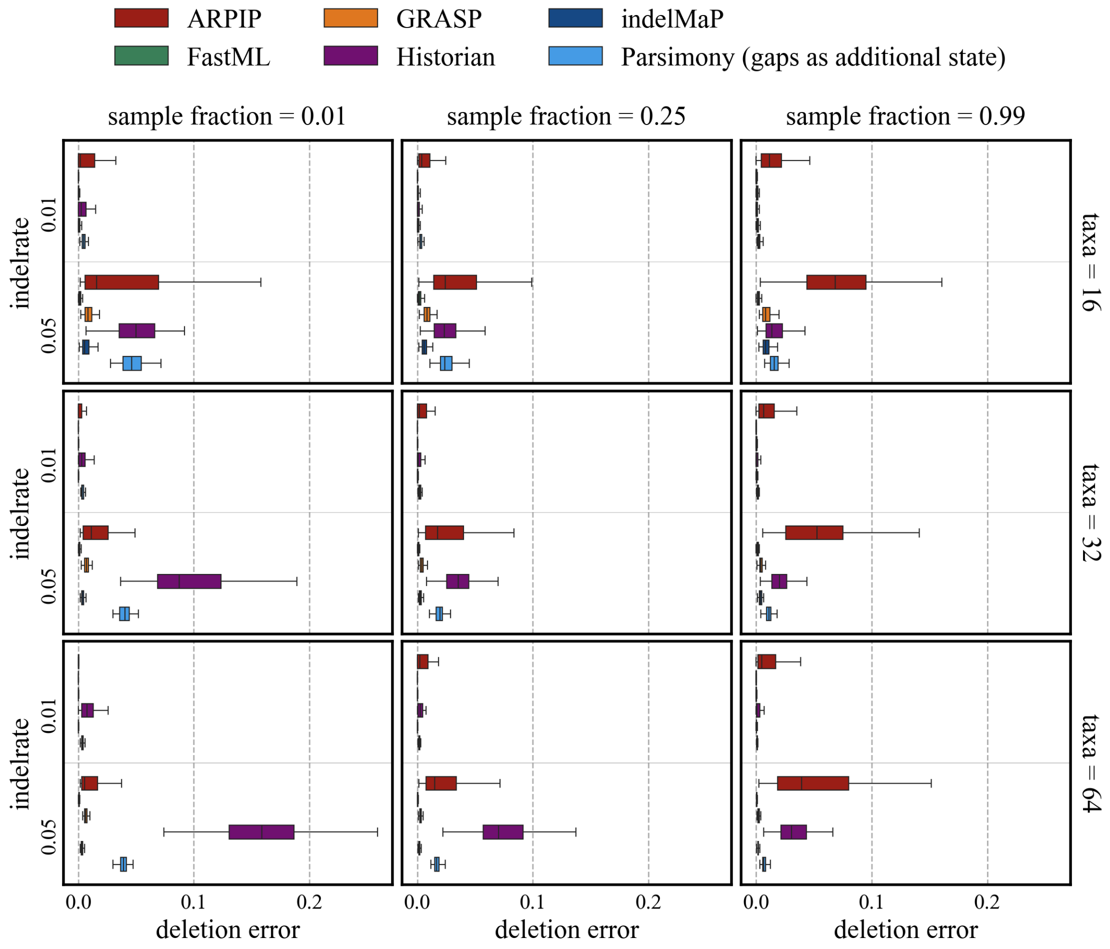

Figure S2: Deletion error for all parameter combinations for trees with tree height 0.8

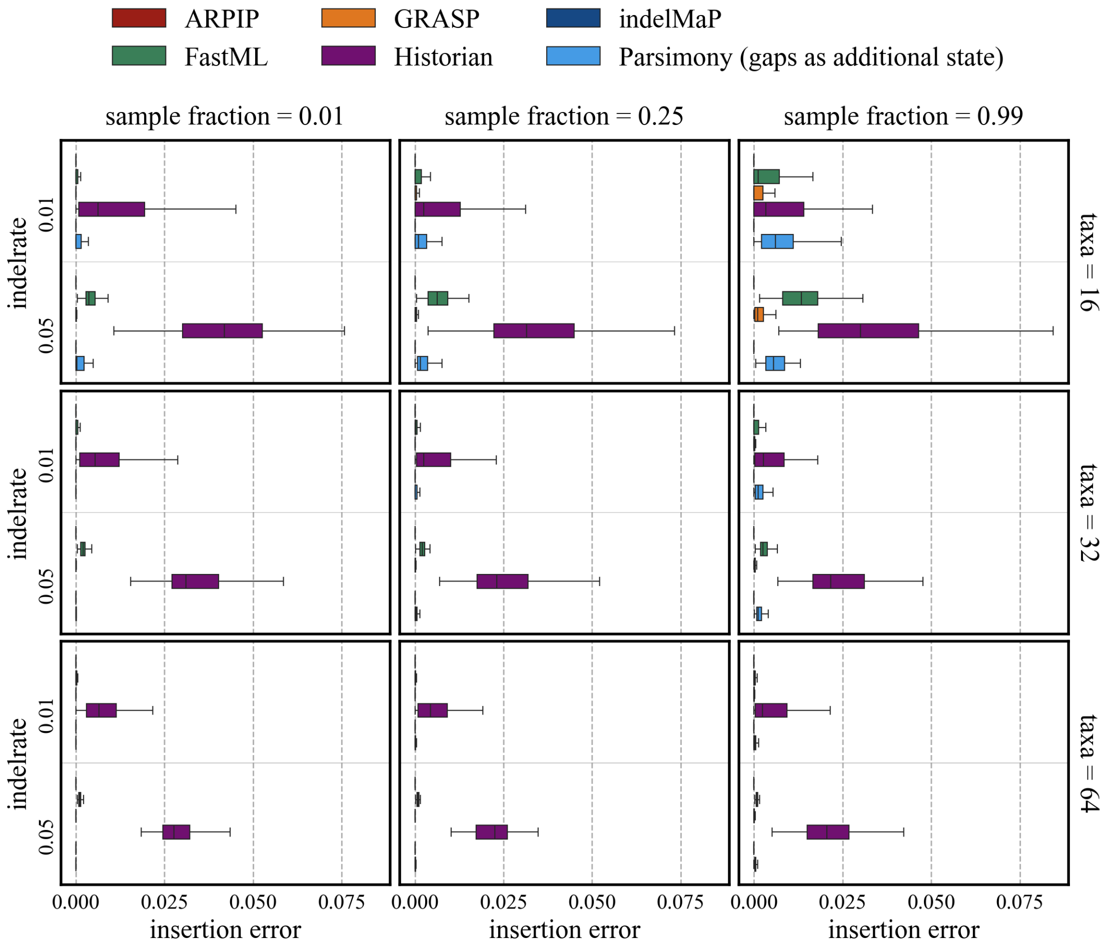

Figure S3: Insertion error for all parameter combinations for trees with tree height 0.8

Ancestral sequence reconstruction accuracy for tree height 1.2 and 1.7

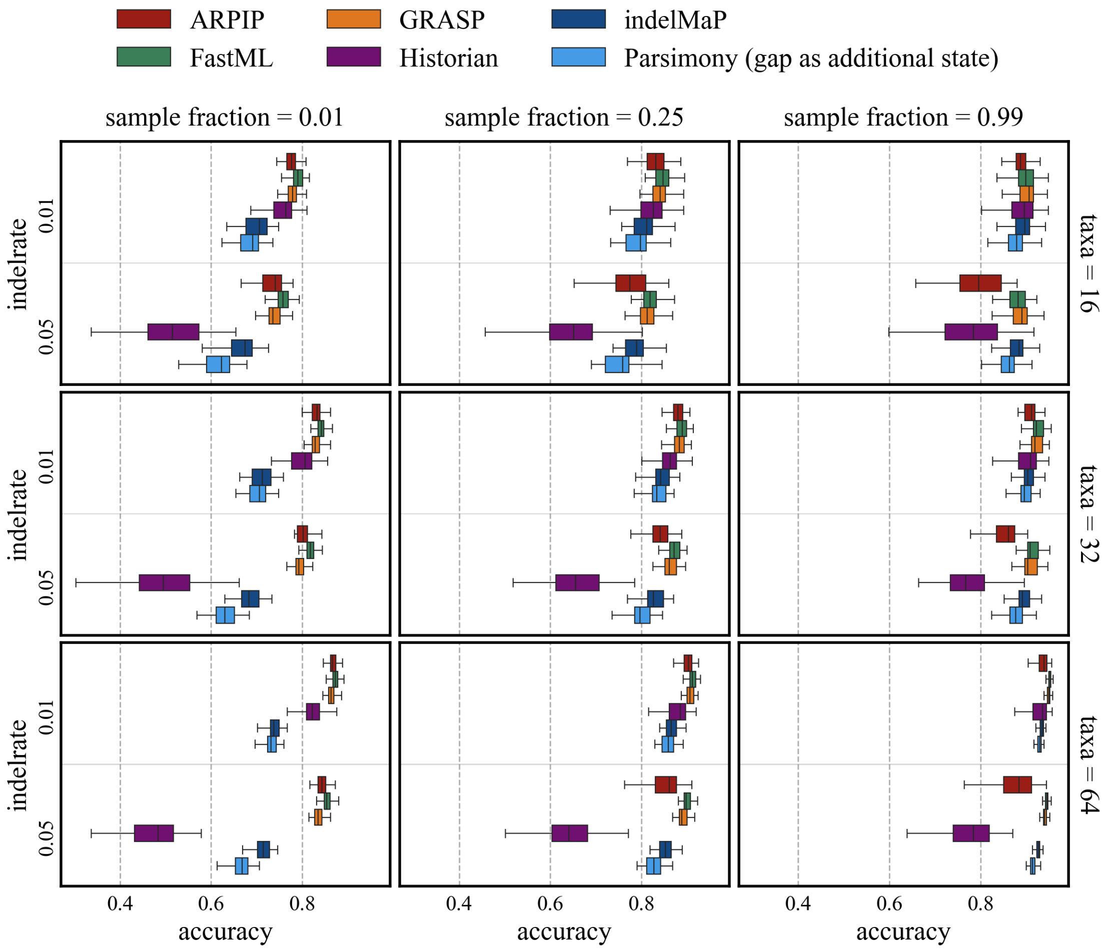

Figure S4: Overall character reconstruction accuracy for all parameter combinations for tree height 1.2.

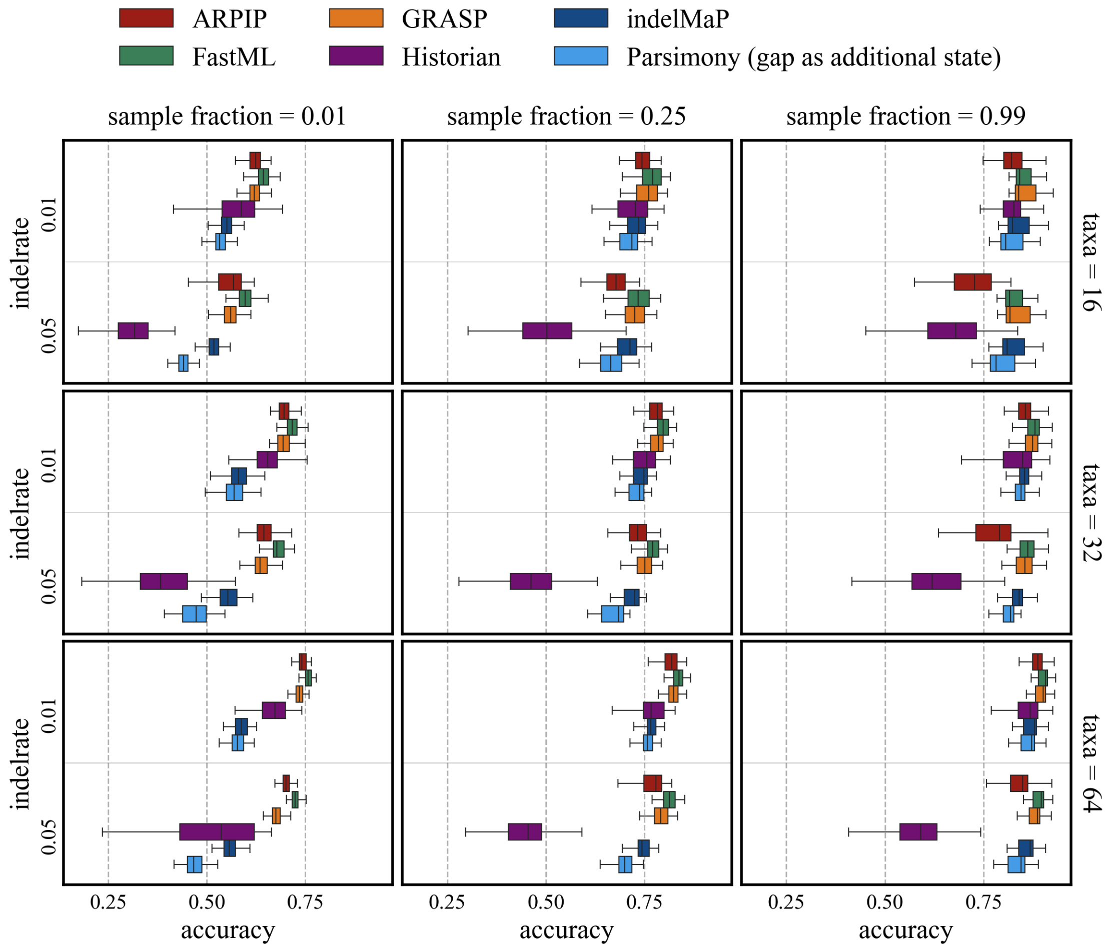

Figure S5: Overall character reconstruction accuracy for all parameter combinations for tree height 1.7.

Ancestral sequence reconstruction substitution, insertion and deletion error for tree height 1.2

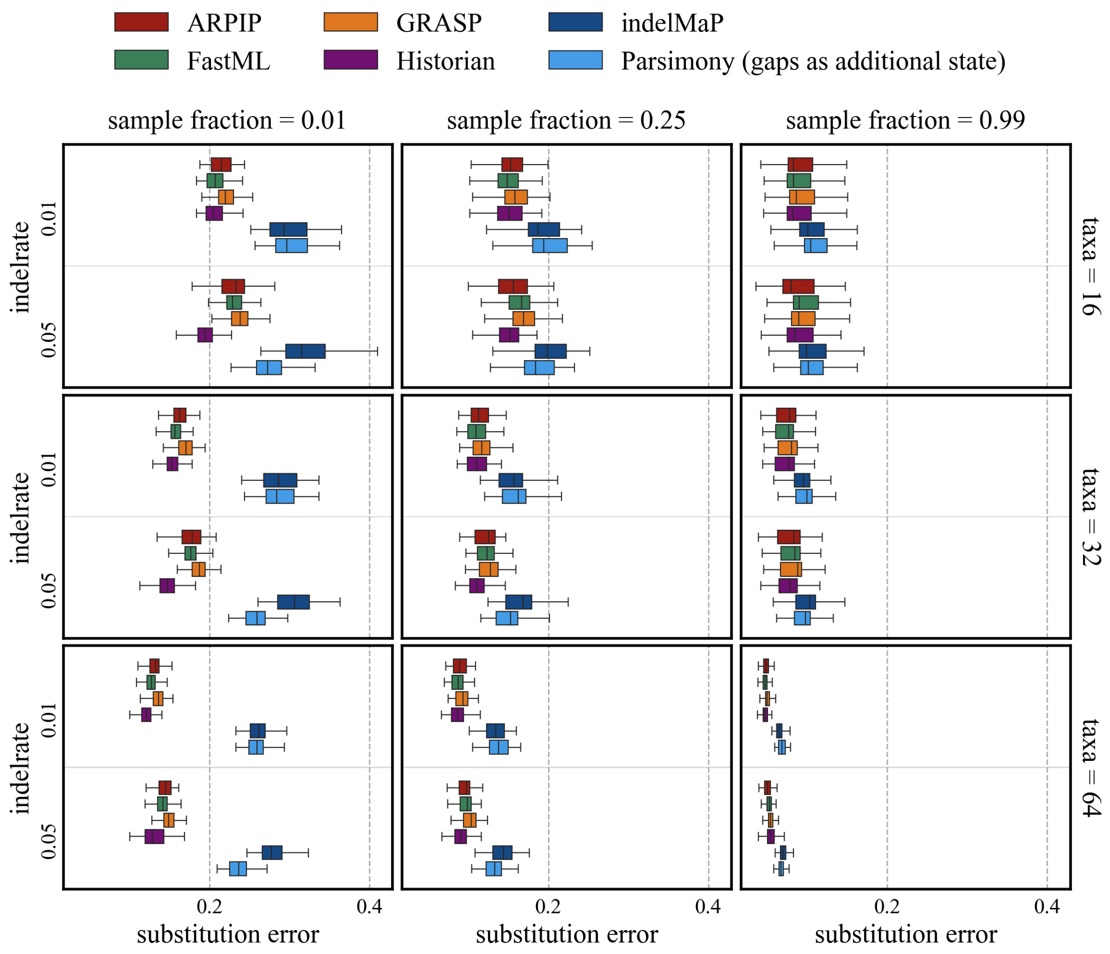

Figure S6: Substitution error for all parameter combinations for trees with tree height 1.2

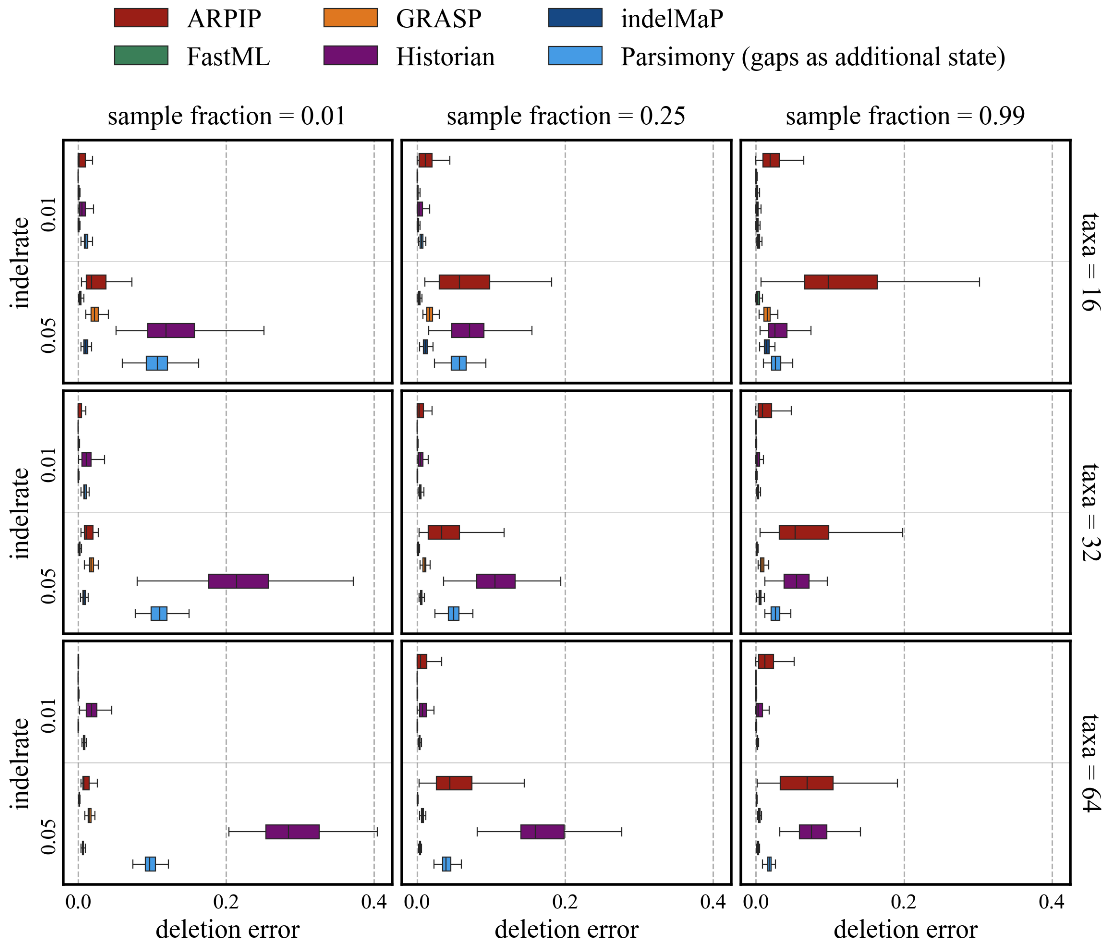

Figure S7: Deletion error for all parameter combinations for trees with tree height 1.2

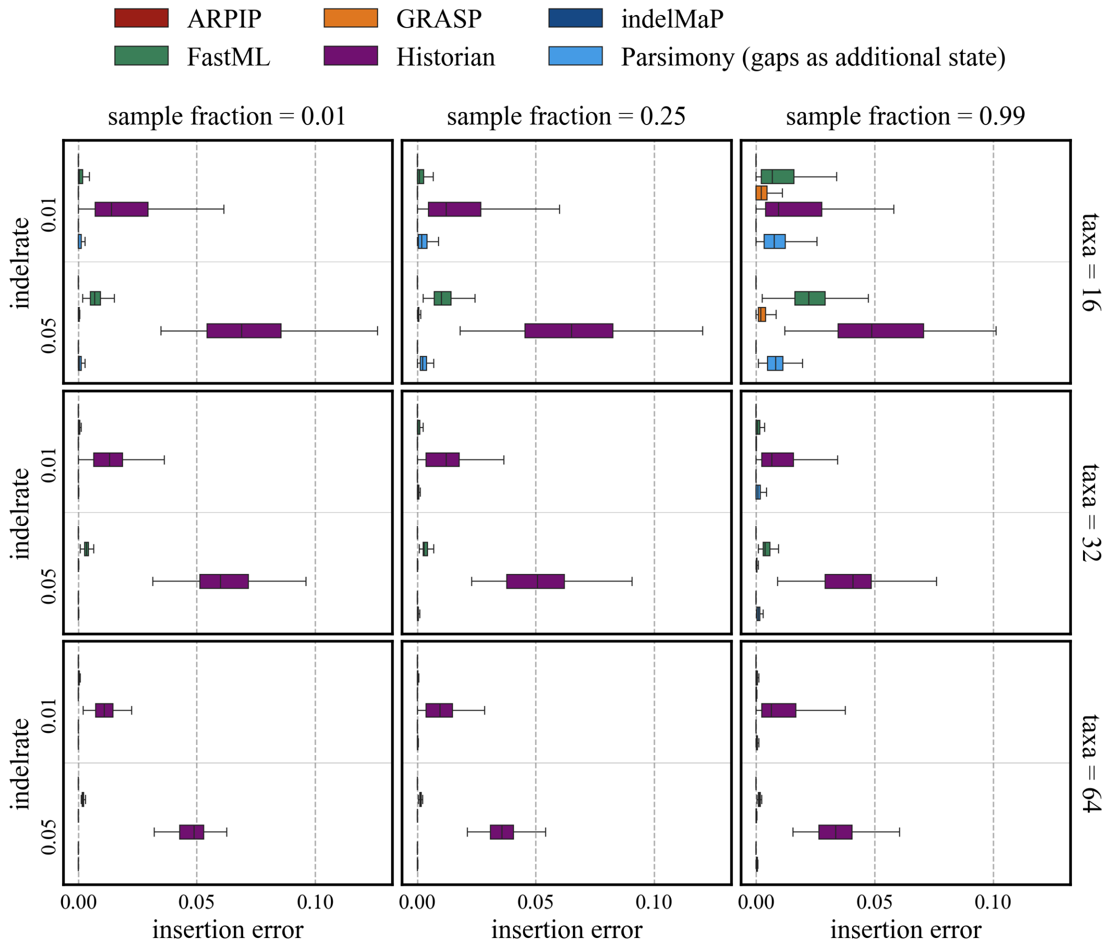

Figure S8: Insertion error for all parameter combinations for trees with tree height 1.2

Ancestral sequence reconstruction substitution, insertion and deletion error for tree height 1.7

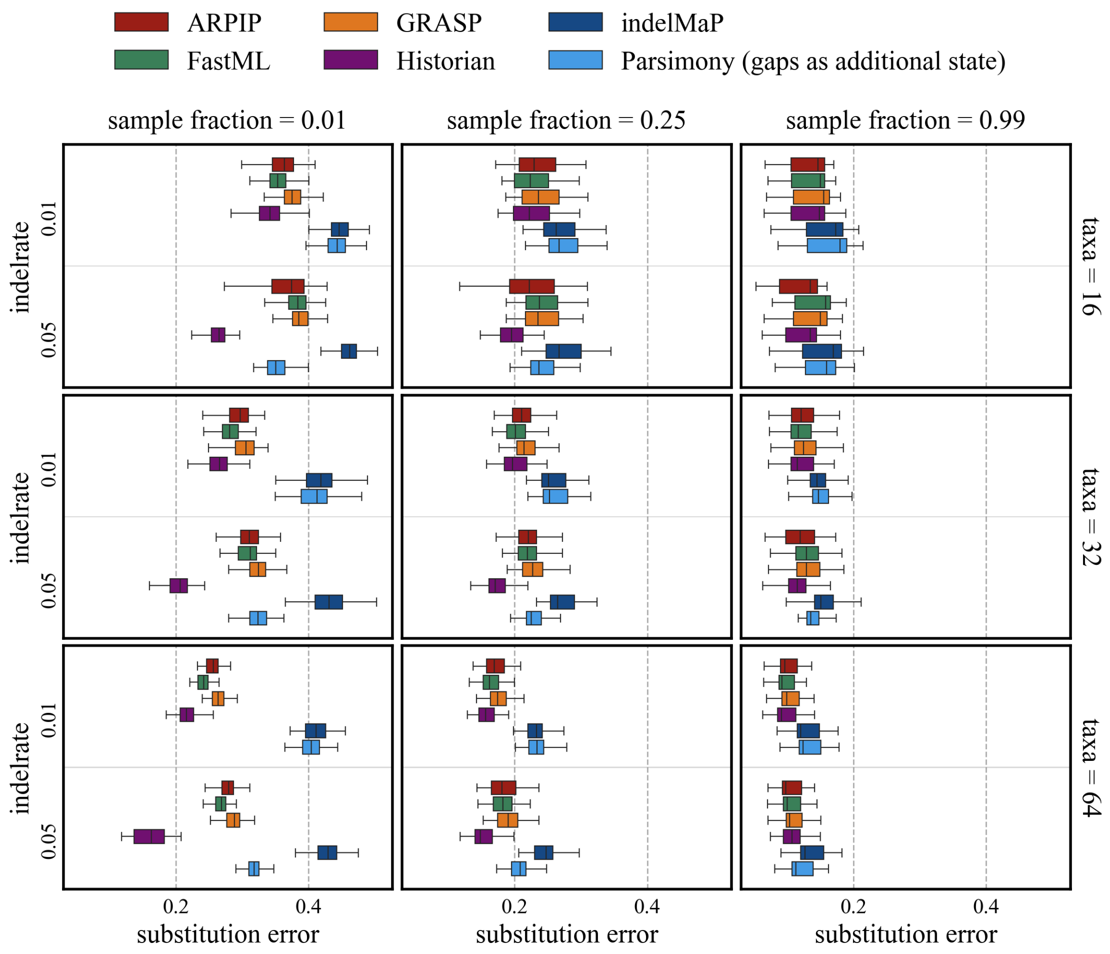

Figure S9: Substitution error for all parameter combinations for trees with tree height 1.7

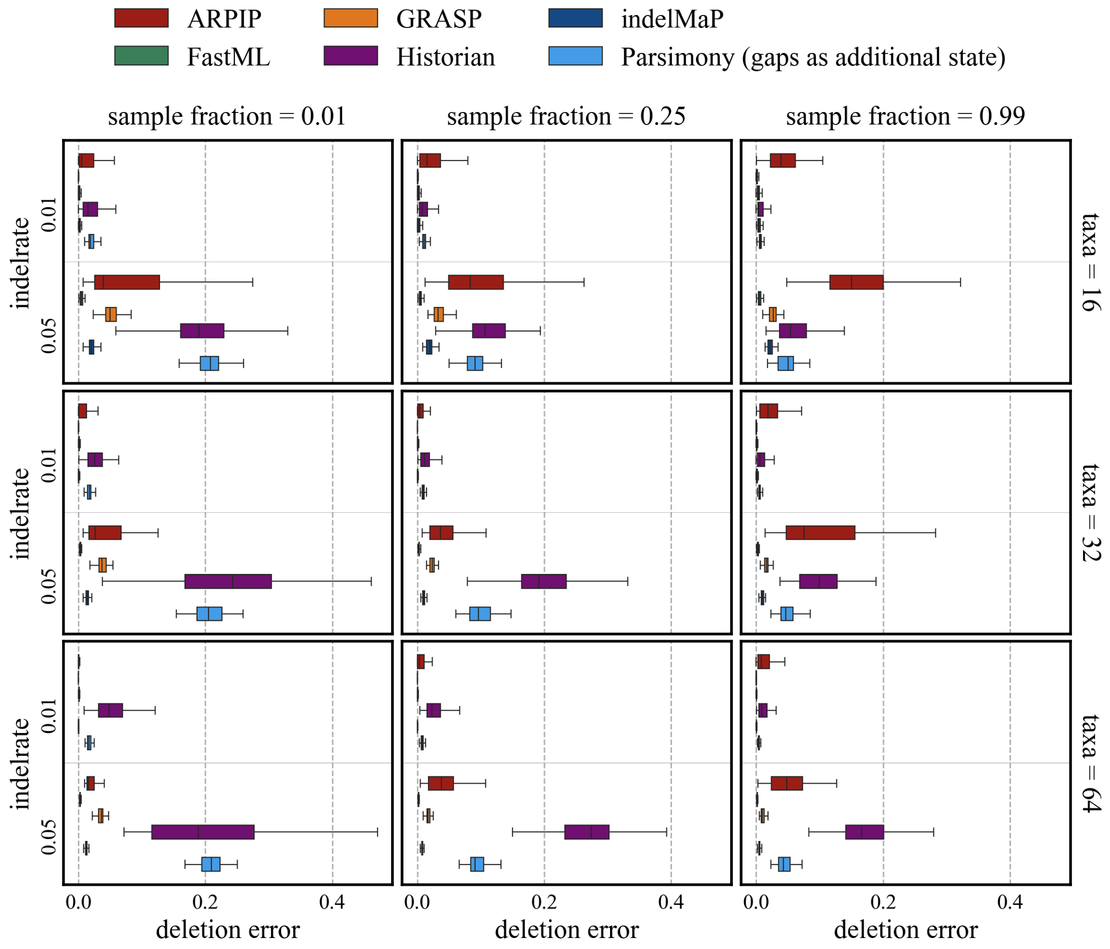

Figure S10: Deletion error for all parameter combinations for trees with tree height 1.7

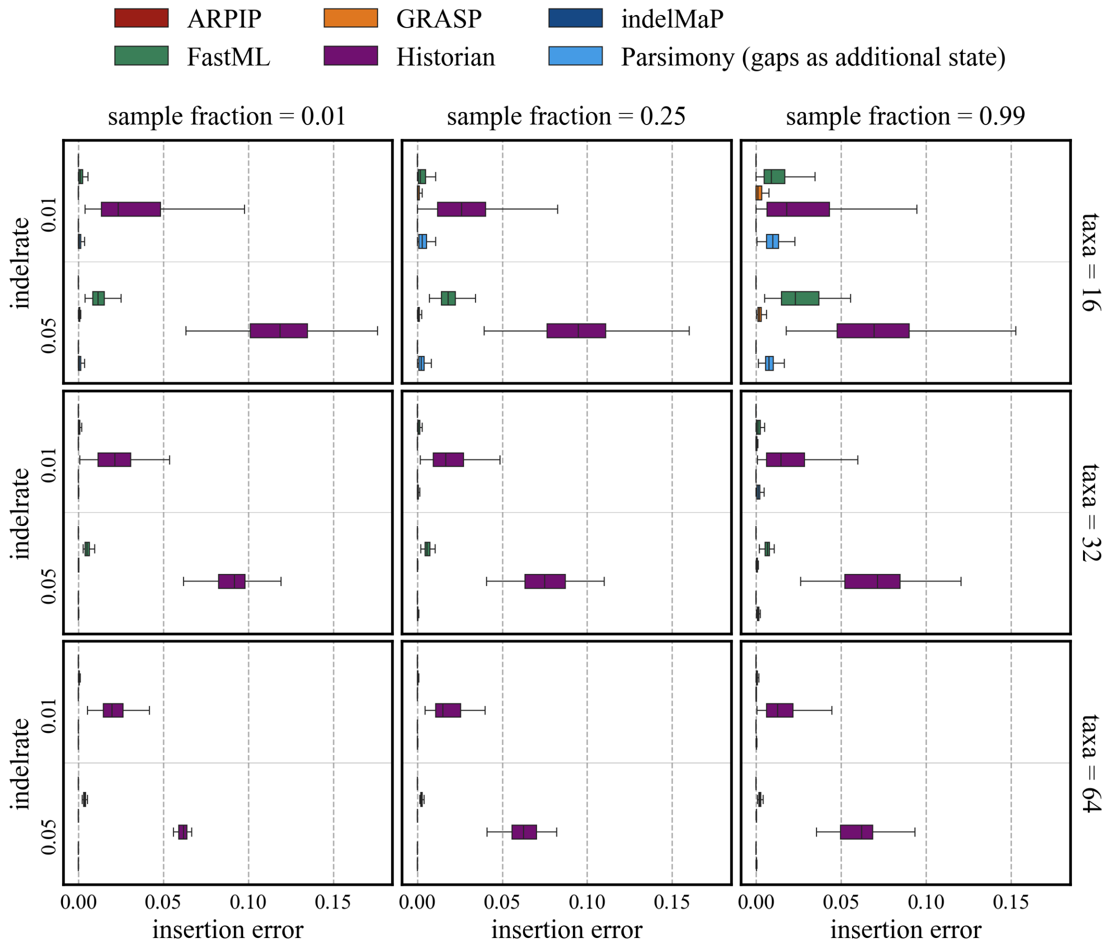

Figure S11: Insertion error for all parameter combinations for trees with tree height 1.7

Correlation between error scores and distance to child nodes

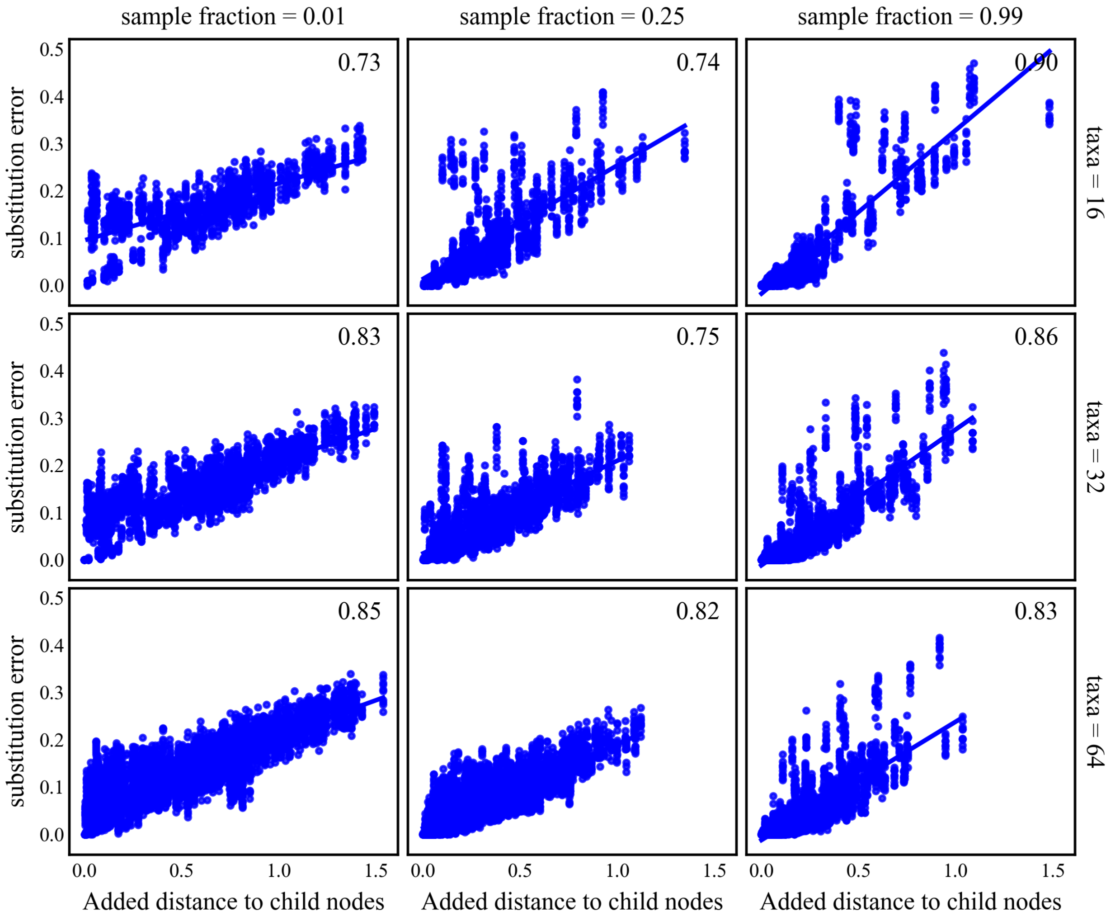

Figure S12: Correlation between added distance to the child nodes and substitution error for all parameter combination with tree height 0.8 and indel rate 0.01.

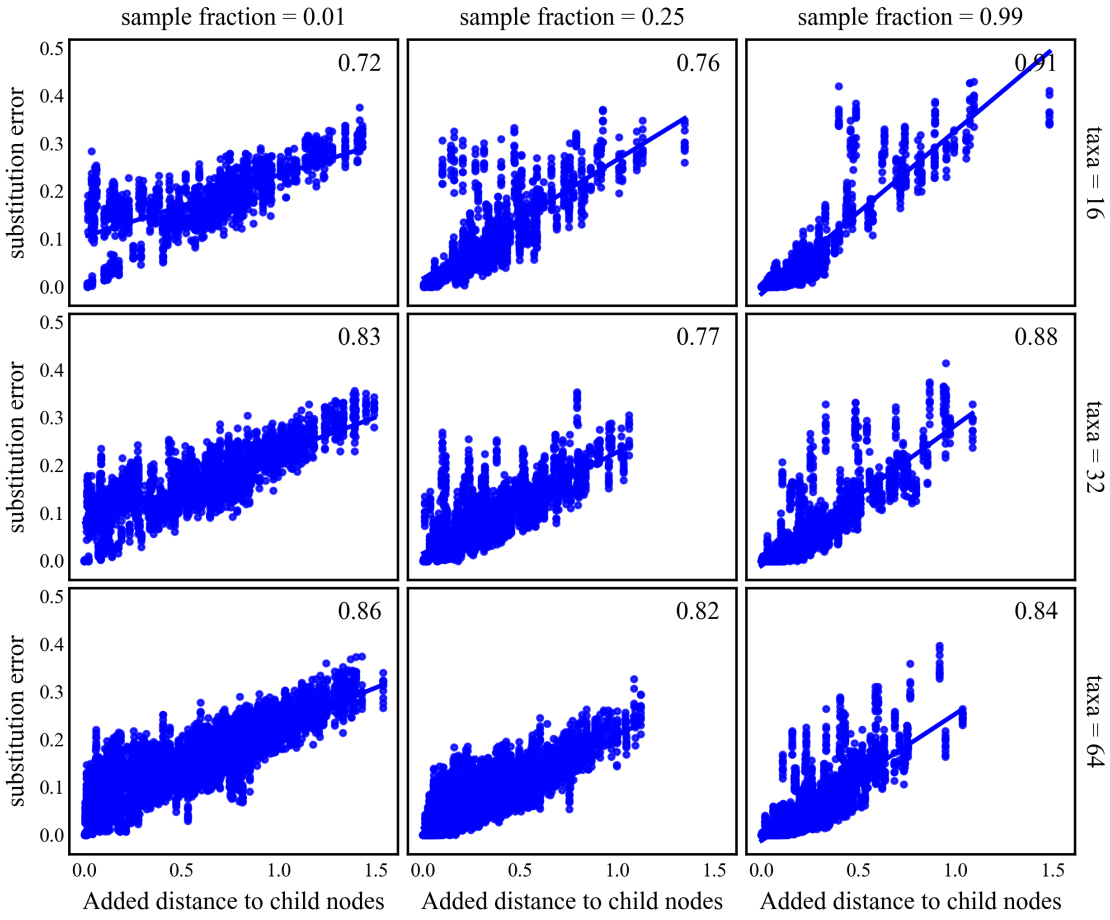

Figure S13: Correlation between added distance to the child nodes and substitution error for all parameter combination with tree height 0.8 and indel rate 0.05.

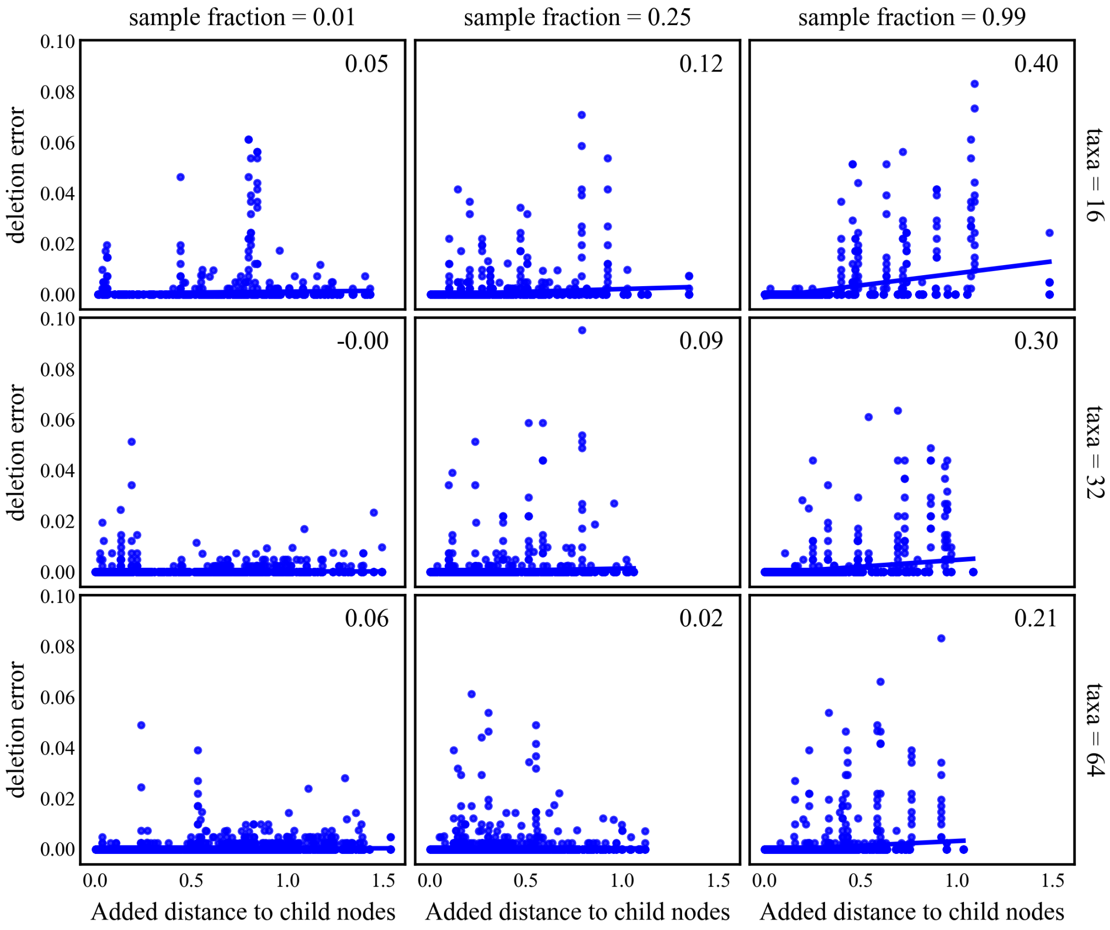

Figure S14: Correlation between added distance to the child nodes and deletion error for all parameter combination with tree height 0.8 and indel rate 0.01.

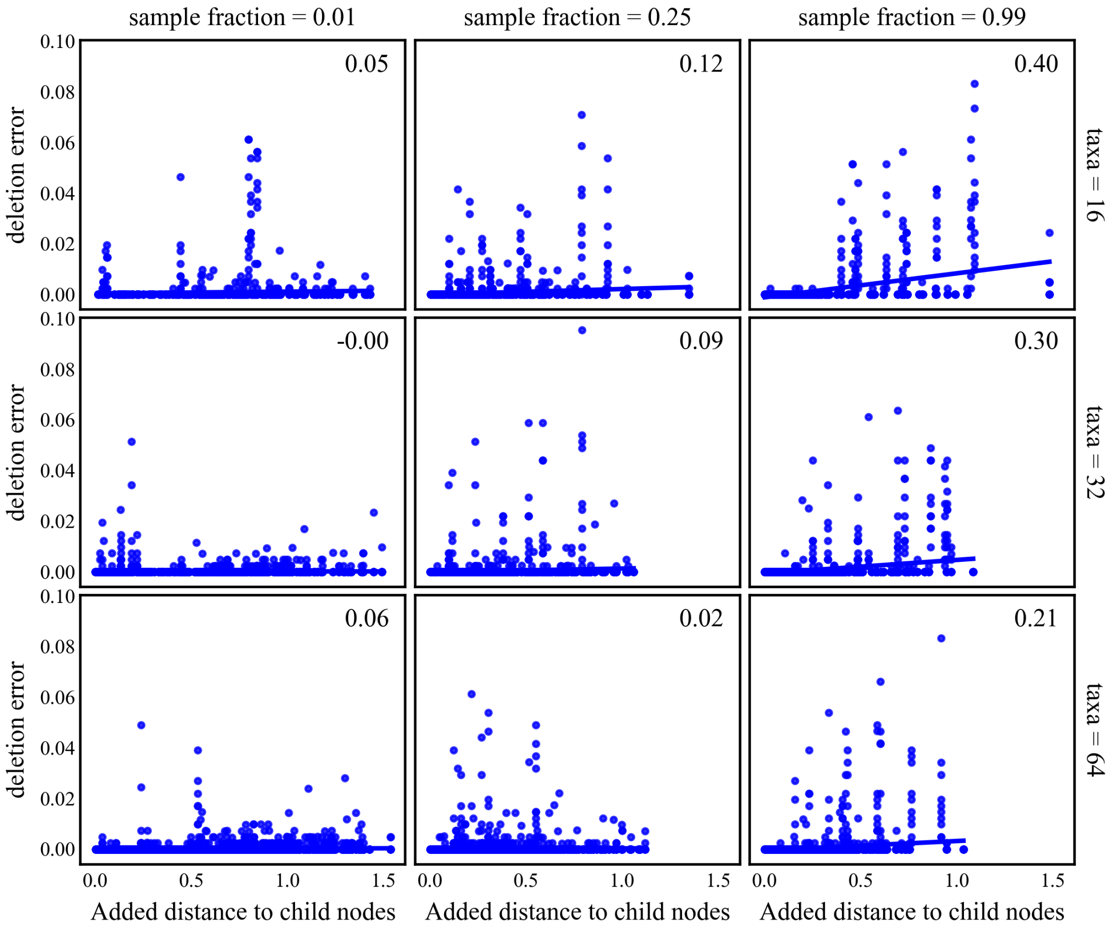

Figure S15: Correlation between added distance to the child nodes and deletion error for all parameter combination with tree height 0.8 and indel rate 0.05.

Multiple sequence alignment quality for tree height 0.8, 1.2 and 1.7

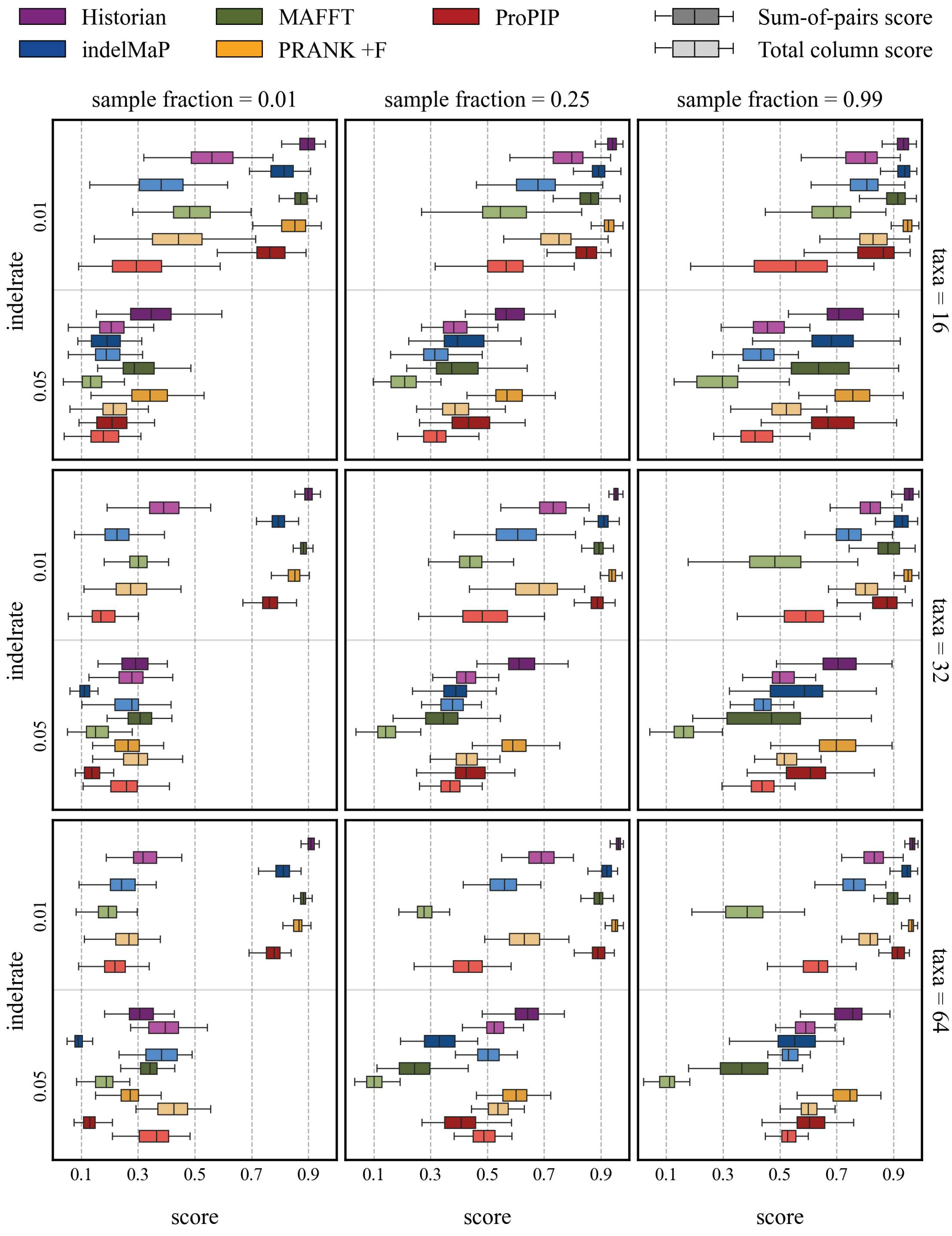

Figure S16: SPSs and TCSs scores for all parameter combinations with tree height 1.2.

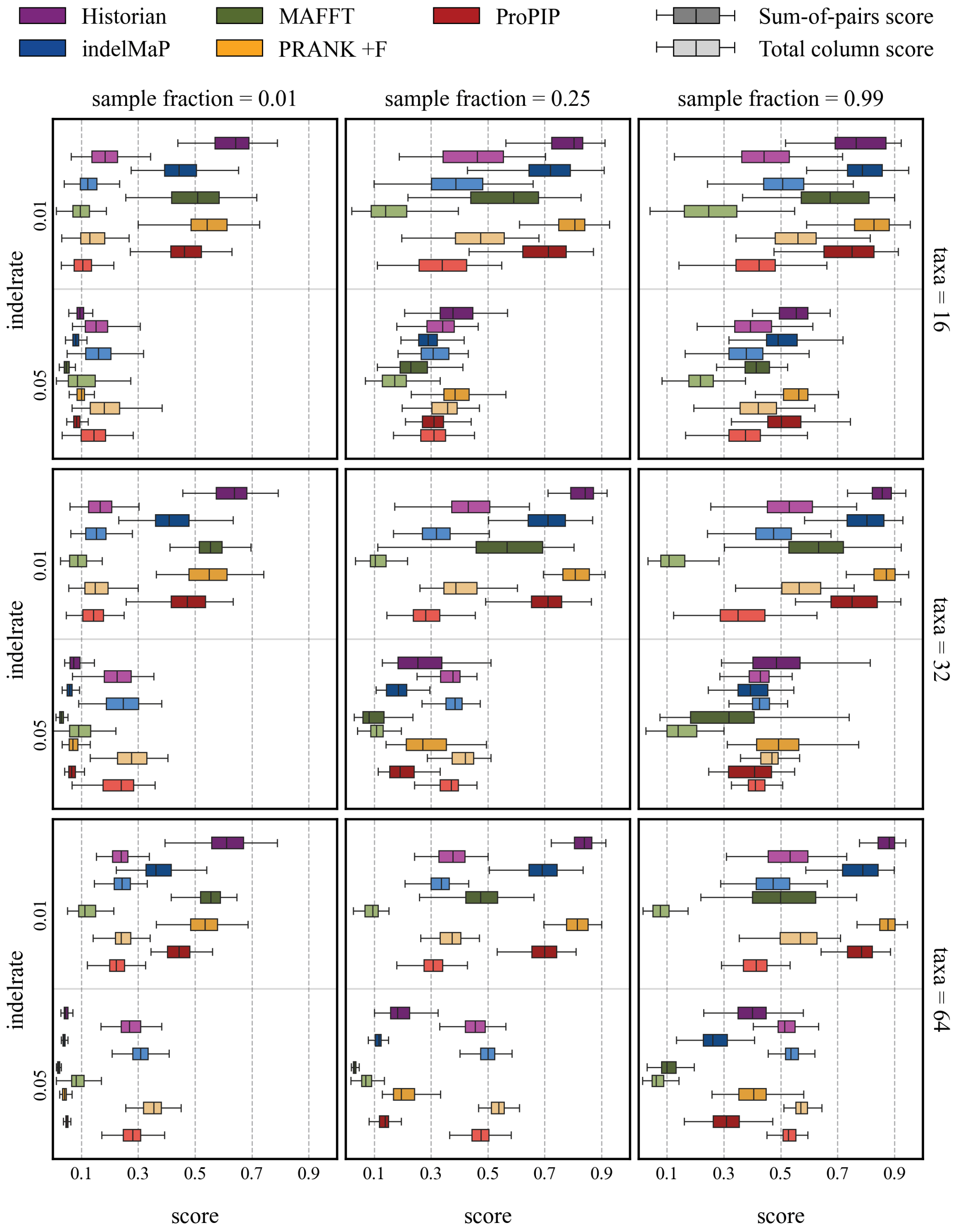

Figure S17: SPSs and TCSs scores for all parameter combinations with tree height 1.7.

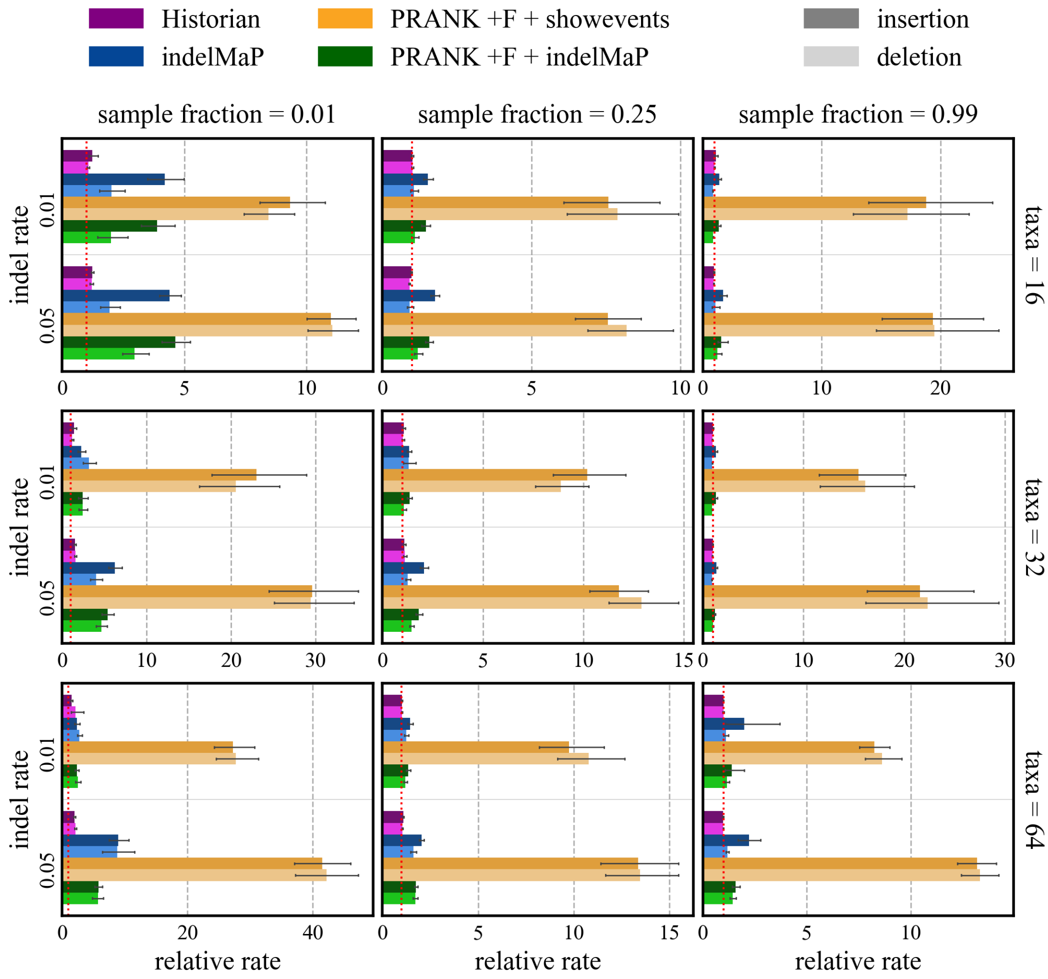

Figure S18: Relative insertion and deletion rates for all parameter combinations with tree height 0.8

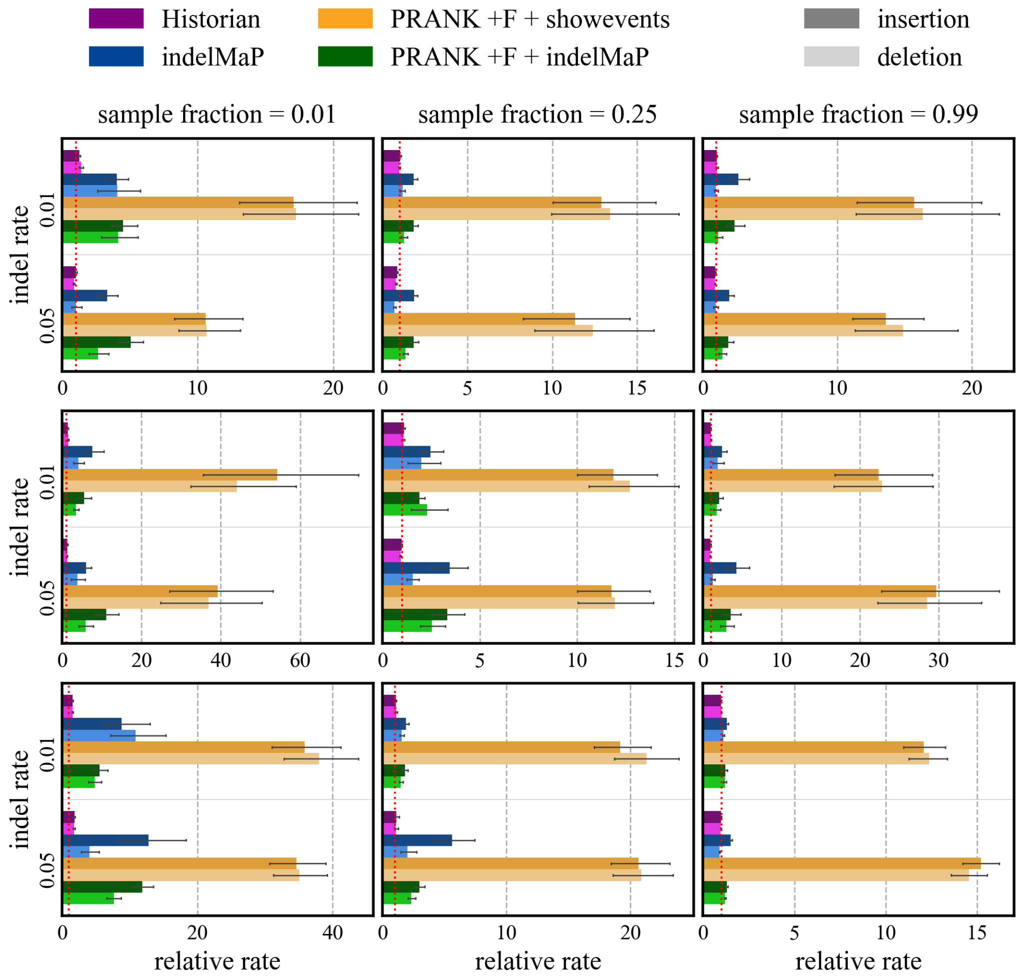

Figure S19: Relative insertion and deletion rates for all parameter combinations with tree height 1.2

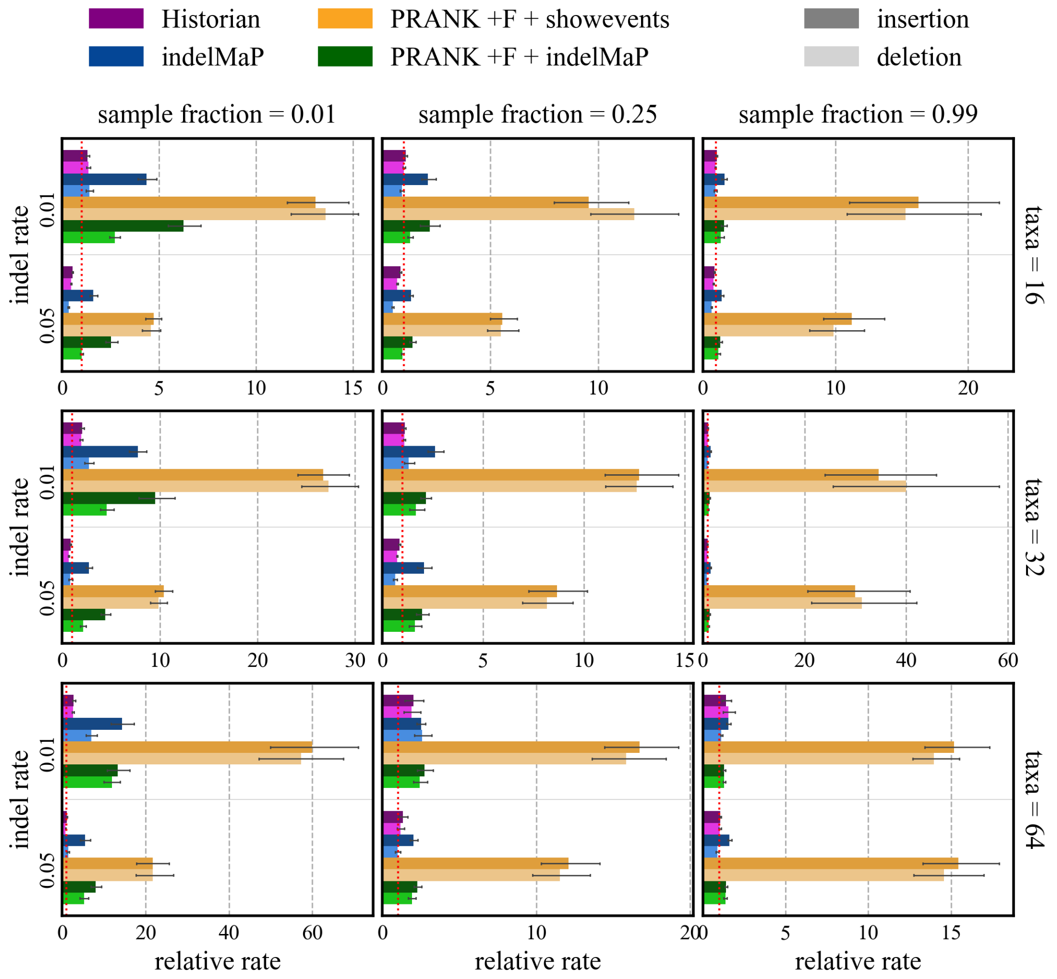

Figure S20: Relative insertion and deletion rates for all parameter combinations with tree height 1.7

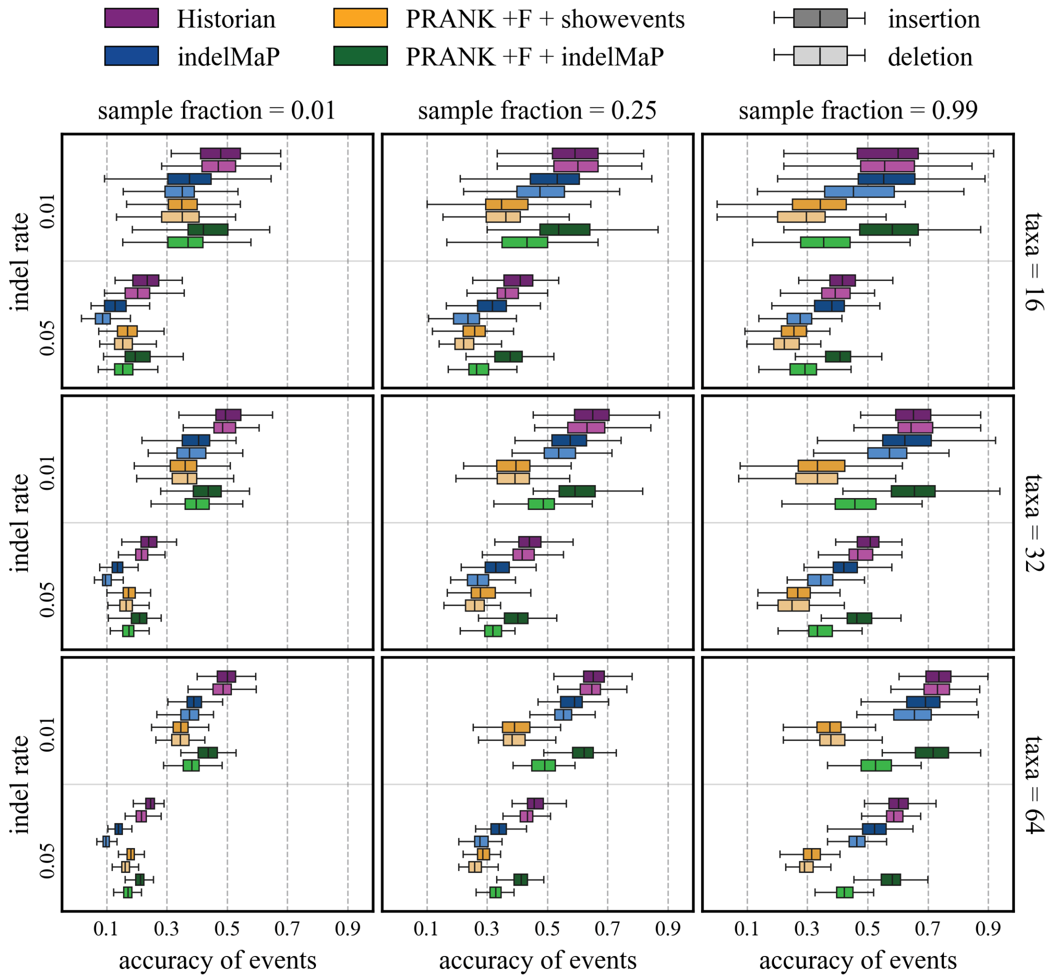

Figure S21: Proportion of accurate inferred insertion and deletion rates for all parameter combinations with tree height 0.8.

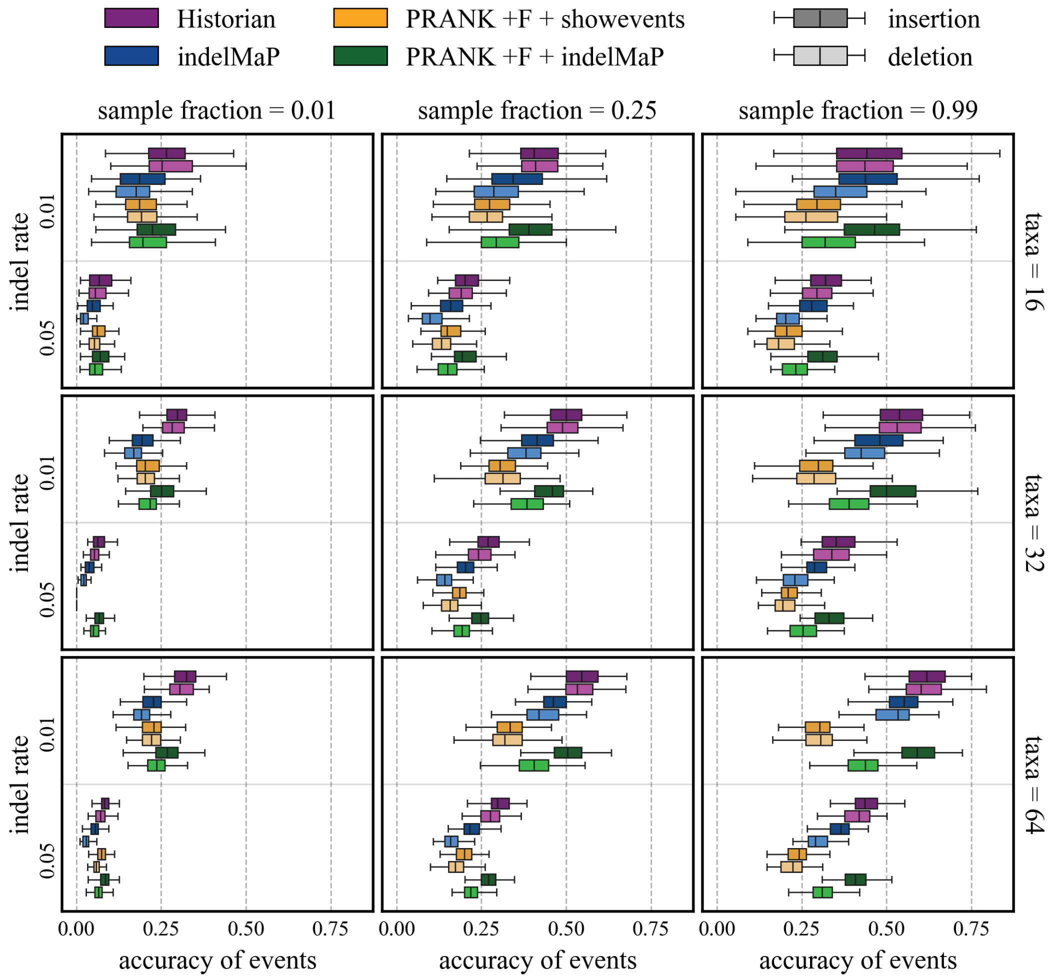

Figure S22: Proportion of accurate inferred insertion and deletion rates for all parameter combinations with tree height 1.2.

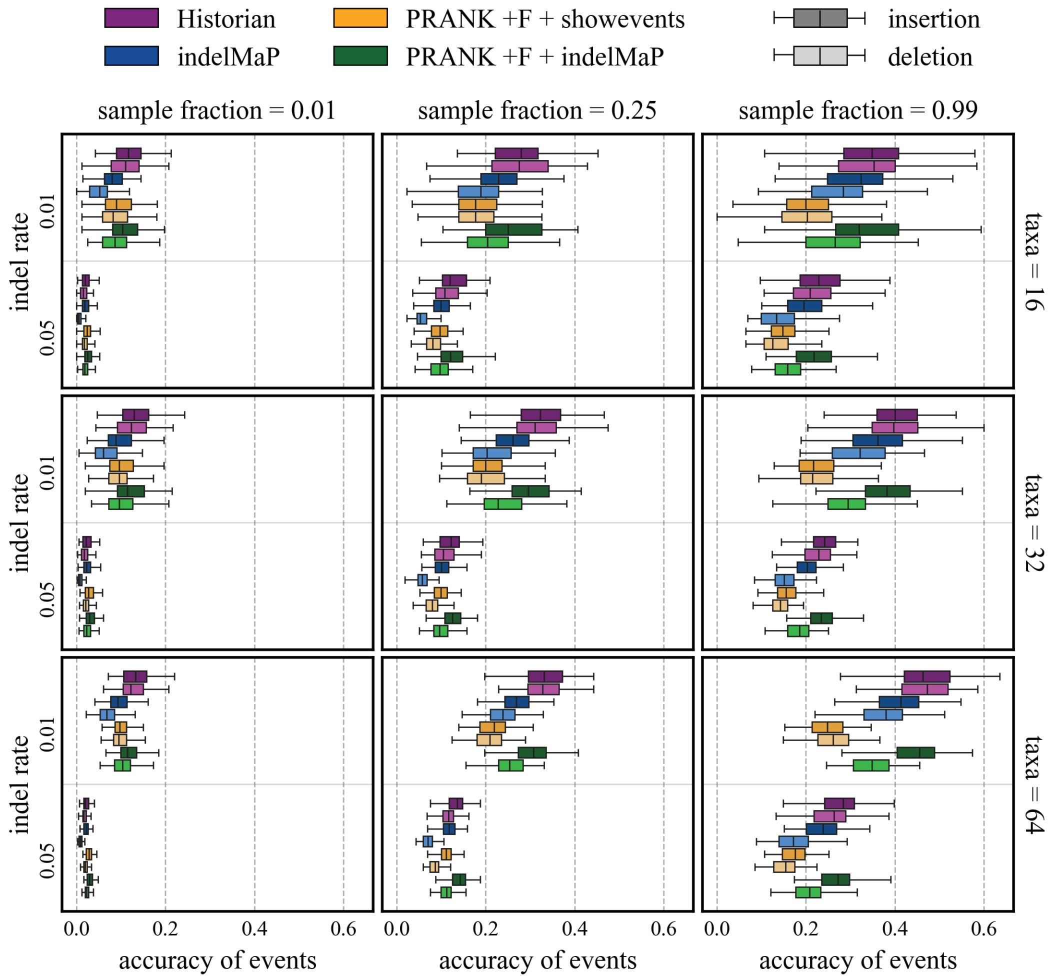

Figure S23: Proportion of accurate inferred insertion and deletion events for all parameter combinations with tree height 1.7.

Multiple sequence alignment quality for estimated guide trees

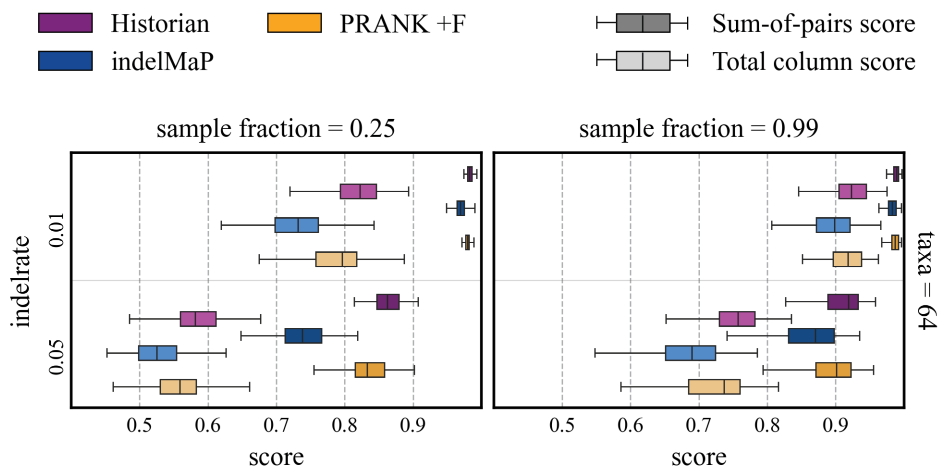

Figure S24: SPSs and TCSs scores for a subset of parameter combinations with tree height 0.8 and 64 taxa. All methods received the same estimated guide tree as guide tree for alignment.

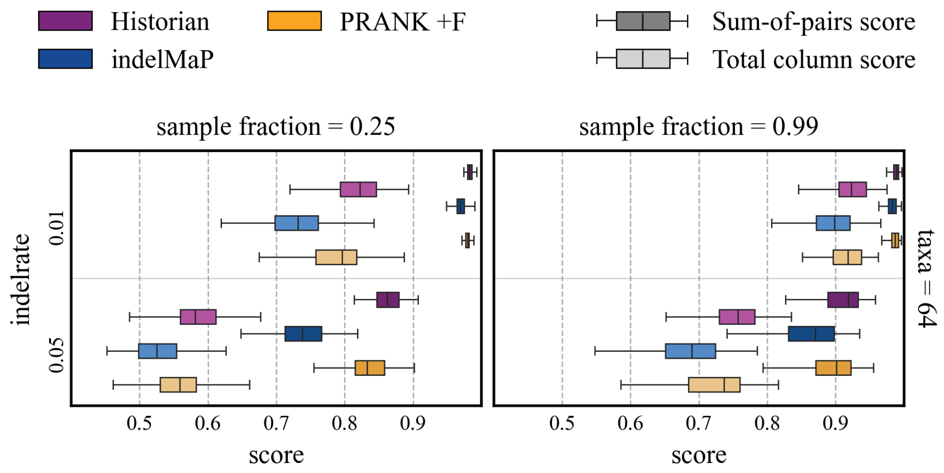

Figure S25: The top figure illustrates a comparison between relative indel rates, specifically the estimated rate over the simulated rate, based on Historian, indelMaP, and PRANK +F. The bottom figure displays the proportion of accurately inferred insertion and deletion events for the same method selection. For PRANK +F we used the events given by the tool and additionally reconstructed ancestors with indelMaP based on the PRANK +F alignment. All methods received the same estimated guide tree.

Sensitivity analysis for gap opening and gap extension penalty

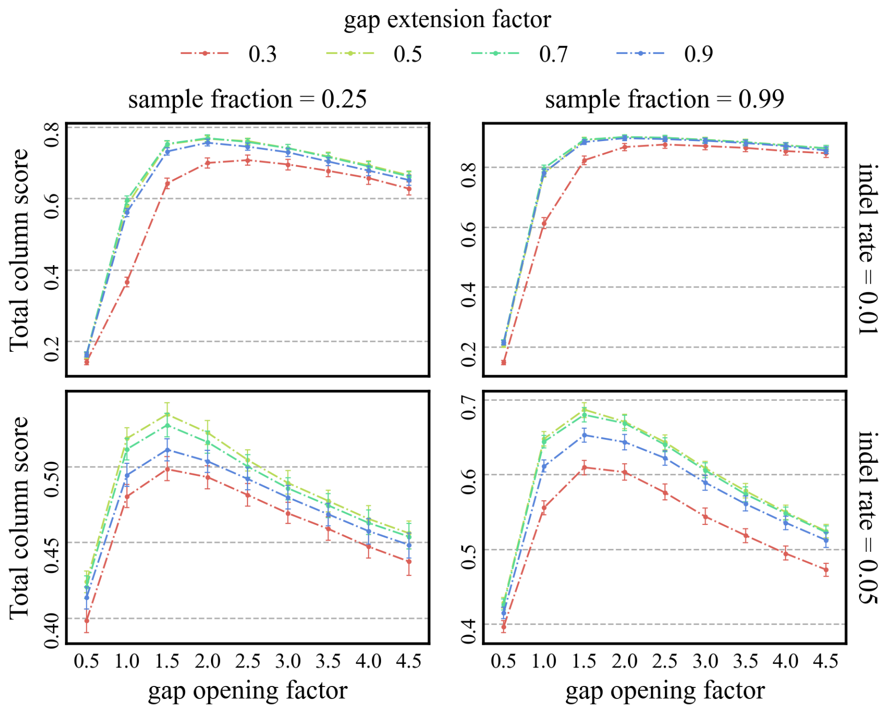

Figure S26: Effect of different combinations of gap opening factor and gap extension factor on the Total column score.

Time benchmark

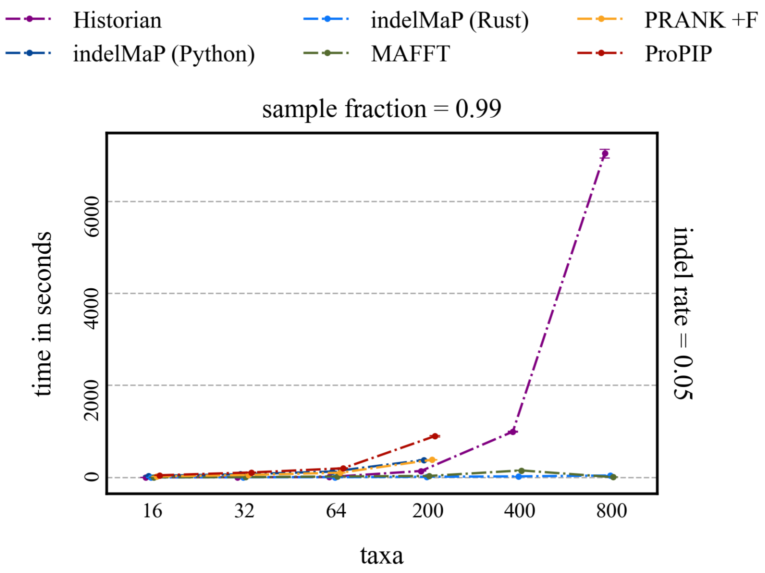

Figure S27: Computational time benchmark of MSA methods, for an extended data set with 400 and 800 taxa trees.

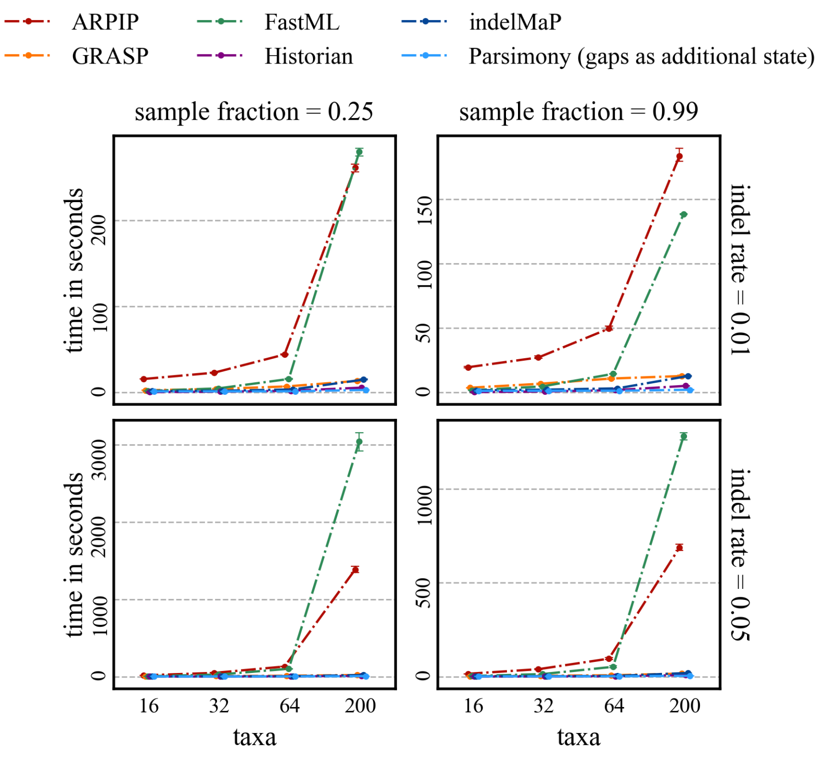

Figure S28: Computational time benchmark of ASR methods, under various parameter combinations with tree height 0.8.

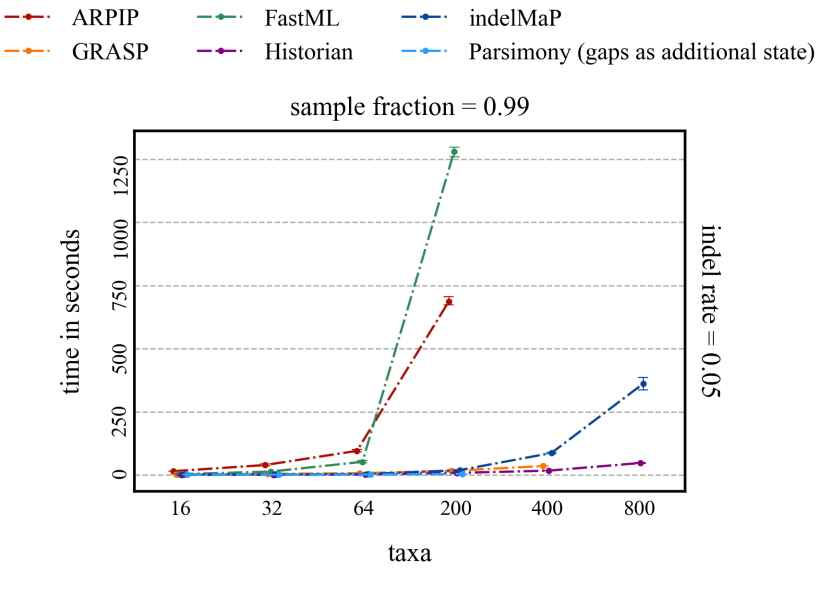

Figure S29: Computational time benchmark of ASR methods, for an extended data set with 400 and 800 taxa trees.

Correlation between log-likelihood under PIP and indelMaP and Parsimony with gaps as an additional state for tree height 1.2

	16 taxa			32 taxa			64 taxa			
	log LK	indelMaP	MP	log LK	indelMaP	MP	log LK	indelMaP	MP	
log LK	-	-0.925	-0.922	-	-0.997	-0.991	-	-0.894	-0.711	**0.25**
indelMaP	-0.986	-	0.978	-0.998	-	0.992	-0.968	-	0.944	
MP	-0.975	0.982	-	-0.99	0.993	-	-0.817	0.927	-	
log LK	-	-0.948	-0.92	-	-0.896	-0.863	-	-0.988	-0.971	**0.99**
indelMaP	-0.983	-	0.993	-0.969	-	0.992	-0.987	-	0.985	
MP	-0.968	0.993	-	-0.942	0.99	-	-0.978	0.992	-	

Table S1: Pearson correlation between the log-likelihood score under PIP (log LK), indelMaP and the conventional parsimony score treating the gap character as an additional state (MP). All parameter combinations are for tree height 0.8.
